# Characterisation of Eco-Innovative Polymer Composites Obtained by Processing Hard-to-Recycle Plastic Waste: Extrusion Parameters, Chemical Composition, and Mechanical Performance

**DOI:** 10.3390/polym18151815

**Published:** 2026-07-24

**Authors:** Tudor Andrei Rusu, Rusu Tiberiu

**Affiliations:** Faculty of Materials and Environmental Engineering, Technical University of Cluj-Napoca, Str. Memorandumului 28, 400114 Cluj-Napoca, Romania

**Keywords:** plastic waste recycling, downcycled mixed plastic, single-screw extrusion, DSC characterisation, mechanical properties, UV weathering, freeze–thaw durability, circular economy, polyethylene, polypropylene, Weber number, CO_2_ mass balance, process optimisation

## Abstract

Problem statement: Contaminated mixed plastic waste—bearing metallic, paper, cardboard and organic residues—remains largely excluded from mechanical recycling because conventional routes require a costly, water- and energy-intensive washing–drying pretreatment. Research gap: No published study combines a fully dry, washing-free valorisation route for such waste with certified mechanical characterisation and a quantified CO_2_ mass balance that explicitly credits elimination of the washing–drying stage. Methodology: This study presents DMP (Downcycled Mixed Plastic), a patented (OSIM, Romania) dry valorisation process based on continuous single-screw extrusion (D = 150 mm, L/D = 17.3), characterised through differential scanning calorimetry (DSC), certified mechanical/thermal testing at accredited Romanian laboratories, Weber-number dispersion analysis, and a process-parameter sensitivity study. Key findings: The composite exhibits certified mechanical properties (tensile strength 9.22 MPa, elongation at break 112.8%, compressive strength 14.5 MPa); composition–property analysis across four batches shows that increasing the PP weight fraction from 20 to 28 wt% raises tensile strength by 8.3% while reducing elongation by 5.2%; a computed Weber number (We = 166.7 ≫ We_crit) is consistent with fine PP-phase dispersion within the PE matrix; the sensitivity study confirms statistically robust structure–property relationships (R^2^ = 0.93–0.98); and the CO_2_ mass balance establishes a net avoidance of 3.150 t CO_2_ eq per tonne of waste processed relative to conventional wet recycling. Significance: dry, washing-free processing is a technically promising pathway for valorising plastic waste streams currently considered non-recyclable, potentially reducing production cost by 60–70% relative to wet recycling, pending additional characterisation identified as priorities for future work.

## 1. Introduction

The accelerating global accumulation of post-consumer plastic waste represents one of the most pressing environmental challenges of the twenty-first century. According to Directive 2006/12/EC of the European Parliament and Council, all EU member states are required to develop waste management plans targeting substantial reductions in the volume of material diverted to landfill and incineration [1]. The European Commission’s Circular Economy Programme (COM (2014) 398 final) mandates a reduction of greenhouse gas emissions by at least 50% by 2030, with binding sanctions for non-compliance [2].

Particular difficulty is encountered in managing heterogeneous, contaminated plastic waste—mixed-polymer streams bearing adhesive labels, food residues or surface coatings—classified as hard-to-recycle under conventional mechanical recycling pathways. Such streams are typically incinerated for thermal energy recovery, generating significant CO_2_ and other greenhouse gas emissions, or deposited in landfill where they persist without biodegradation [3].

Polyethylene (PE, (C_2_H_4_)_n_) and polypropylene (PP, (C_3_H_6_)_n_) are the dominant polymer families in municipal plastic waste. PE is produced in structural variants including LDPE (density 0.910–0.940 g/cm^3^), LLDPE, and HDPE (~0.97 g/cm^3^) [4]. PP exhibits a melting point of approximately 160–171 °C for the isotactic homopolymer and 200–280 °C for copolymers and homopolymers [5].

An emerging approach to the valorisation of mixed, contaminated plastic waste involves melt-blending and extrusion of the commingled stream into metal moulds to produce semi-finished structural products—a process that avoids costly washing and sorting operations. The resulting material, termed downcycled mixed plastic (DMP), has been proposed for urban infrastructure and construction applications [6]. However, a rigorous multi-method characterisation of DMP composites produced by continuous single-screw extrusion—encompassing differential scanning calorimetry (DSC), tensile and compressive behaviour, durability under accelerated weathering, bending, wear, water absorption and thermal conductivity—has not previously been reported for composites of this specific composition and process origin.

Recent systematic reviews confirm that contamination remains the primary technical barrier limiting the scale-up of mechanical recycling: mixed and contaminated plastic streams require additional sorting and cleaning steps that increase energy use and reduce process profitability [7]. Reviews of hazardous and mixed plastic waste streams similarly identify contamination by mixed polymers and organic residues as the primary factor degrading recyclate quality and driving up the resource intensity of the sorting–washing sequence [8]. A large-scale, industrial-facility sampling campaign directly comparing source-separated and post-sorted (mixed-waste) plastic packaging streams found that, although target-polymer purity is broadly comparable between the two collection routes, post-sorted material consistently carries higher levels of moisture, dirt, volatile organic compounds and restricted trace metals, confirming that recovering plastics from mixed, contaminated waste increases available feedstock but does so at a systematic quality and purification-cost penalty that washing does not fully offset [9]. Earlier work on collection-system comparisons similarly found that contamination and performance variability remain considerable across separation systems, even where sorting infrastructure is well developed [10].

Where mixed polymer fractions cannot be avoided, compatibilisation strategies have been developed to recover mechanical performance. Reactive extrusion using maleic-anhydride-grafted or glycidyl-methacrylate-based compatibilisers has been shown to substantially improve interfacial adhesion and mechanical properties in immiscible PP/PET and PP/rPET systems [11,12]. Copolymer-based compatibiliser design has more broadly been framed as a route to reduce interfacial tension and increase interfacial strength across mixed-plastic waste streams of varying composition [13], while simulation-guided extrusion process design combined with PP-g-MAH compatibilisation has been used to improve dispersion of recycled PP/PET nanocomposite blends [14]. Critically, these compatibilisation studies have been developed and validated primarily on pre-sorted, washed polyolefin or polyolefin/PET feedstocks—a precondition that the washing-free process addressed in the present work does not assume. This distinction is central to the present study: interfacial dispersion quality is established here as a primary lever for mechanical performance in a contaminated, unwashed feedstock, which motivates the mechanistic (Weber-number-based) treatment of PP-phase dispersion adopted in Section 2.5, in place of a compatibilisation strategy validated only for cleaner input streams.

A review of prior patent literature further confirms this gap. Patents describing recycling routes for plastic waste into moulded or pelletised products [15,16] both require a washing and drying step prior to extrusion. A patent disclosing a polyolefin blend with enhanced surface durability [17] is formulated from virgin or pre-sorted polypropylene-based resins rather than contaminated waste streams. A patent disclosing a polypropylene-based extruded foam process [18] starts from a defined polypropylene resin rather than a heterogeneous, contamination-bearing waste blend. None of these four representative patents addresses a fully dry, washing-free process applied directly to plastic waste contaminated with metals, paper, cardboard and organic matter, reinforcing the novelty claim made below from the standpoint of the patent literature as well as the peer-reviewed literature.

No published study, to the authors’ knowledge based on the literature reviewed above, combines a fully dry, washing-free valorisation route for contaminated mixed plastic waste with certified multi-condition mechanical characterisation and a complete, quantified CO_2_ mass balance that explicitly credits the elimination of the washing–drying stage. This gap follows directly from three specific deficiencies in the existing literature: (i) contamination is repeatedly identified as the structural bottleneck of mechanical recycling, yet the resource-intensive washing–drying pretreatment it necessitates is treated as a fixed cost rather than a variable that can itself be eliminated and credited; (ii) compatibilisation and processing strategies capable of recovering mechanical performance in heterogeneous polyolefin blends have not been validated on feedstocks still bearing metallic, paper, cardboard and organic contamination; and (iii) CO_2_ accounting of recycling routes has not isolated the specific climate benefit of eliminating the washing–drying stage as a distinct, quantified term.

Building on this gap, the present study makes four specific, delineated contributions, stated here explicitly to avoid overstating originality beyond what is defensible: (i) a fully dry, washing-free valorisation process (OSIM patent pending) applied directly to contaminated, non-homogeneous plastic waste, in contrast to established mixed-polyolefin extrusion routes that rely on pre-sorted, washed feedstocks; (ii) a mechanistic, Weber-number-based explanation of the resulting composite morphology (Section 2.5); (iii) a process-parameter sensitivity study establishing statistically robust structure–property relationships across the operating envelope (Section 2.6 and Section 3.4); and (iv) a complete CO_2_ mass balance that explicitly isolates and credits the climate benefit of eliminating the washing–drying stage (Section 4.6). Mixed PE/PP waste extrusion and recycled-plastic construction profiles are not, in themselves, novel; the specific contribution of this work lies in the combination of these four elements applied to a contaminated, washing-free feedstock stream.

**Figure 1:**
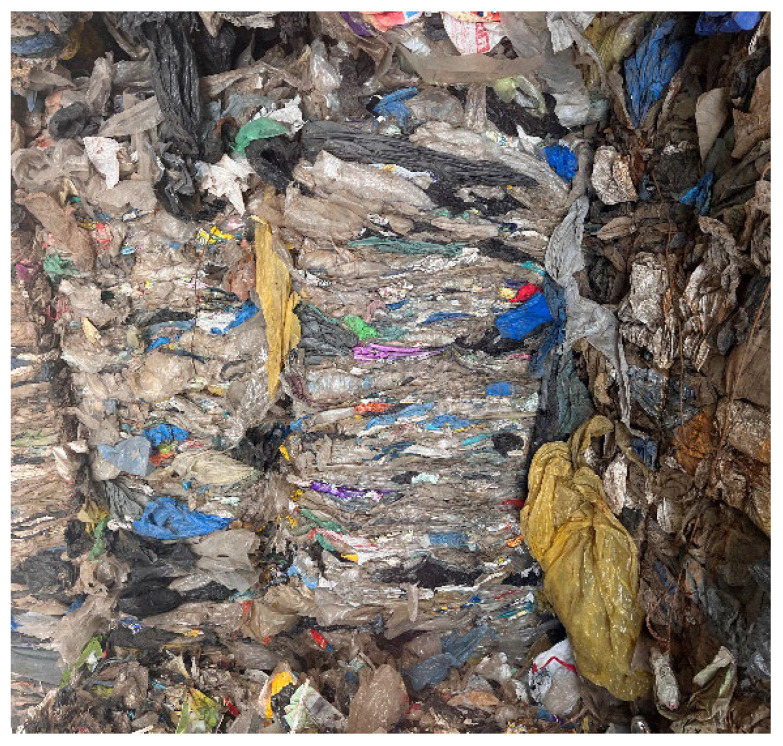
Contaminated, mixed post-consumer plastic waste (hard-to-recycle stream) prior to comminution and extrusion processing.

This characterisation was produced under the technical and scientific supervision of Prof. Rusu Tiberiu by Tudor Andrei Rusu at the Technical University of Cluj-Napoca, in collaboration with S.C. DMP Manufacture Innovation S.R.L. (Timisoara, Romania). All characterisation was performed by accredited laboratories: ICECON S.A. (Bucharest); URBAN-INCERC National Institute; Babes-Bolyai University ICCRR (Cluj-Napoca); ECOIND; and the Faculty of Civil Engineering, Technical University of Cluj-Napoca. Figure 1 shows a representative sample of the contaminated, mixed post-consumer plastic waste stream processed in this study.

## 2. Materials and Methods

### 2.1. Raw Material Composition

The raw material consists of heterogeneous post-consumer plastic waste classified as hard-to-recycle: mixed, contaminated packaging foils and rigid packaging items bearing adhesive labels, food residues or surface coatings. Non-polymeric contamination (paper/cardboard, textiles, wood, metals, and organic matter) accounts for approximately 2 wt% of the incoming feedstock. Prior to processing, the waste undergoes the following: (1) manual sorting and selection; (2) size reduction (comminution) to a particle size of d50 = 2–8 mm by knife milling; and (3) dry decontamination by compressed-air blowing to remove loose dust and fine debris. No washing, aqueous cleaning or drying stage is applied at any point, as elimination of this pretreatment is the central process innovation of the DMP route; no additional mixing step beyond the compressed-air decontamination is performed. PET is present in the waste stream at approximately 5–10 wt%, in part in fibrous form arising from the contraction of PET strip fragments (e.g., from packaging straps) during the melting stage in the extruder, where it functions as a reinforcing fibrous phase within the composite (Section 4.2). Extruder feed rate, post-moulding cooling rate, detailed die/nozzle geometry and specimen orientation relative to melt flow direction were not systematically recorded during the characterisation campaign reported in this manuscript and are identified as a limitation (Section 4.9).

Polymer weight fractions were determined by manual sorting and gravimetric weighing of visually and texturally identified fractions from each waste batch (typical sample mass 2–5 kg per batch, balance precision ± 1 g), with polymer identity assigned by reference to literature melting-point ranges (Section 2.2) rather than by an independent spectroscopic method; this sorting-based gravimetric protocol, and its implications, are discussed as a methodological limitation in Section 4.9. Compositional analysis of seven independently received waste batches established the aggregate weight-fraction ranges reported above (60–70 wt% PE, 20–30 wt% PP, 5–10 wt% other polymers, ~2 wt% impurities). Batch-to-batch variability across these seven deliveries was, respectively, PE 60–70 wt% (mean ≈ 65 wt%), PP 20–30 wt% (mean ≈ 24 wt%), and other polymers 5–10 wt% (mean ≈ 8 wt%), indicating a feedstock composition that is consistent in broad polymer class but variable at the individual-batch level—a variability further reflected in the four characterised batches L-1 to L-4 (Section 3.3: 68/22, 62/28, 70/20 and 65/25 wt% PE/PP, respectively). The 2 wt% non-polymeric impurity level represents an aggregate figure across paper/cardboard, textiles, wood, metals and organic matter, established during process development and consistent with the patent documentation underlying the DMP process; it was not measured independently per batch or broken down by individual contaminant category, which is identified as a limitation (Section 4.9). In response to reviewer feedback on this point, we note that the batches characterised in this study were drawn from S.C. DMP Manufacture Innovation S.R.L.’s existing pilot-scale testing schedule rather than from samples specifically reserved for additional independent analysis, so no spectroscopic or other independent compositional method could be applied retrospectively to the material examined here; commissioning an independent compositional method (e.g., FTIR or a systematic density-separation protocol) on a newly reserved batch set has been accordingly adopted as the immediate priority for the next characterisation campaign (Section 4.9).

**Figure 2:**
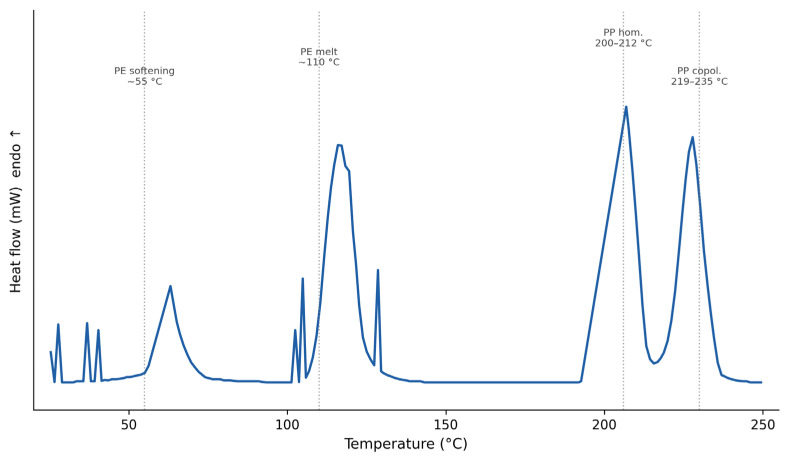
Schematic illustration of the characteristic differential scanning calorimetry (DSC) thermal transitions for the DMP composite, constructed from the tabulated values in Table 1: PE (softening ~55 °C, melt ~110 °C), PP homopolymer (melt 200–212 °C, glass transition ~230 °C), and PP copolymer (melt 219–235 °C) fractions, based on Samples 1–3 (Mettler-Toledo 823ᵉ, 10 °C/min, N_2_; Babes-Bolyai University ICCRR, Contract No. 82/21.01.2025). Samples 4 and 6, which show substantially different transition temperatures, are not represented in this schematic and are discussed separately (Section 3.1 and Section 4.9). The upward arrow on the heat-flow axis indicates the endothermic direction.

DSC analysis of five representative samples was performed at the Babes-Bolyai University Institute for Research in Chemistry RALUCA RIPAN (Cluj-Napoca) under Contract No. 82/21.01.2025, using a Mettler-Toledo 823ᵉ Calorimeter (Mettler-Toledo, Greifensee, Switzerland). Measurements were conducted in the temperature interval 25–250 °C under a nitrogen protective atmosphere, with an aluminium crucible (40 μL, lidded), heating rate 10 °C/min and a final isothermal plateau of 0.5 min. Figure 2 shows the schematic DSC thermogram.

**Table 1:** DSC characterisation of representative DMP composite samples (Mettler-Toledo 823ᵉ, 10 °C/min, N_2_, ICCRR/UBB Contract No. 82/21.01.2025).

Sample	Mass (mg)	T Range (°C)	Softening T (°C)	Melting T (°C)	Tg (°C)	Tcrist (°C)	Polymer
1	10.724	25–80	55	–	–	–	PE
		80–120	110	–	–	–	
		120–210	–	203	–	–	PP copol.
		210–230	–	219	–	–	
		230–250	–	235	–	–	
3	8.966	25–80	50	–	–	–	PP hom.
		80–205	–	200	–	–	
		205–225	–	212	–	–	
		225–250	–	–	230	–	
2	6.774	25–120	114	–	–	–	PE
		120–250	–	167	–	–	PP
4	11.350	25–120	108	–	–	–	PE
		120–135	125	–	–	–	
		135–185	–	169	–	–	PP
		185–250	–	–	–	214	
6	13.280	25–190	–	165/168	–	–	PP
		190–300	–	–	–	257/284	

Softening T = softening temperature; Melting T = melting peak temperature; Tg = glass transition temperature; Tcrist = crystallisation temperature. All five samples submitted under Contract No. 82/21 January 2025 are reported (Samples 1–4, 6; no Sample 5 was submitted).

### 2.2. Extrusion Equipment and Process Parameters

Processing was carried out using a continuous single-screw extrusion line. The facility operates three identical lines, each consisting of the following: (1) a central control panel; (2) a pneumatic granule conveying and metering system; (3) the main plasticating/extrusion unit; (4) a water-cooled calibration and cooling section; (5) a haul-off unit; and (6) a cross-head cutter.

The single-screw extruder is equipped with a screw of diameter D = 150 mm and length L = 2600 mm, giving L/D = 17.3. The screw rotational speed is maintained at 30 rpm. The barrel temperature profile is set at 200 °C (feed/compression zone), rising to 220 °C (metering/discharge zone). Metal moulds (dies) are independently heated to 80–120 °C. Extrusion pressure varies with die geometry: 60–80 MPa for simple cross-section profiles and 100–120 MPa for thin-walled components [19].

### 2.3. Product Range and Applications

**Figure 3:**
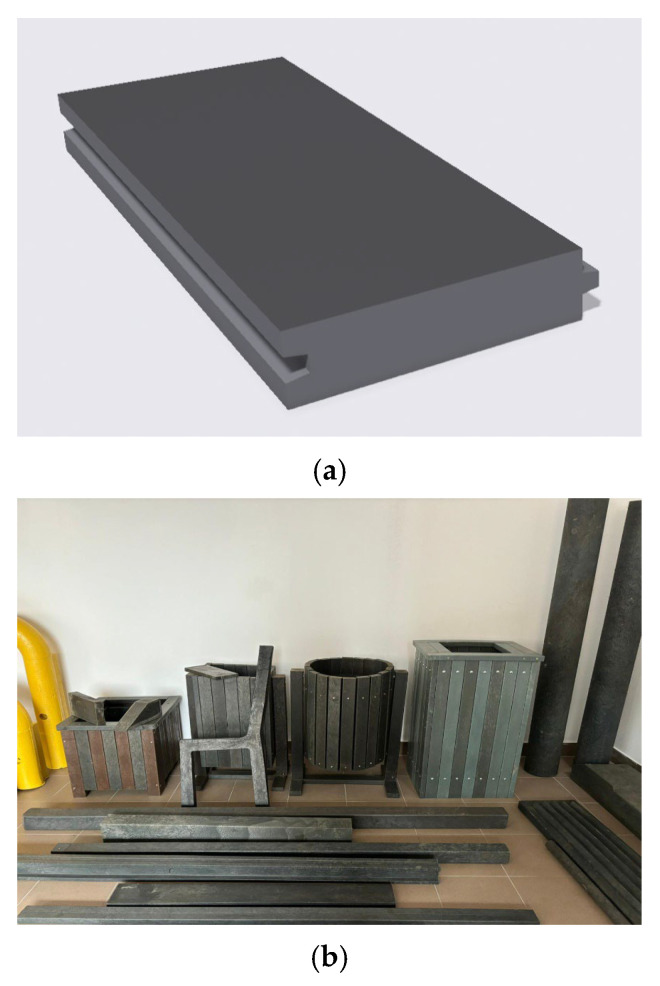
DMP composite products: (**a**) parking slab; (**b**) extruded traffic-management profiles. Products exhibit dark grey colour characteristic of mixed-waste feedstock.

The DMP composite is extruded into a wide range of profiles for construction and urban infrastructure applications, including parking slabs, column/wall protection profiles, urban furniture components and traffic-management profiles. The material can be cut, drilled, bonded and mechanically fastened. Figure 3 shows representative DMP products.

### 2.4. Test Specimen Preparation

Tensile specimens were prepared in accordance with SR EN ISO 527-2:2012 (Type B flat dumbbell geometry). Specimens were conditioned at 23 ± 2 °C and 50 ± 5% relative humidity for a minimum of 88 h prior to testing, in compliance with SR EN ISO 291.

The standards applied in this study were selected as follows. SR/EN ISO 527-1:2020 and SR/EN ISO 527-2:2012 were used as the standard tensile-testing methods for rigid plastics. SR EN ISO 1183-1:2019 (Method A, water immersion) was used as the standard method for plastics density. SR EN 477:2018 (drop-height/energy method) was used as the relevant standard for impact resistance of construction profiles. SR EN 12390-3:2019 (compressive strength) and the SR EN 998-2 minimum reference threshold for masonry mortar were applied because the DMP composite is benchmarked, for its intended lightweight construction application, against concrete and masonry mortar as comparator materials (Section 4.4); this benchmarking choice does not imply that DMP is itself a concrete or mortar product. SR EN 12839:2012 and SR EN 12839:2018 were applied as the relevant standards for bending load and horizontal load resistance of free-standing construction/urban-furniture profiles, consistent with the product applications described in Section 2.3.

The durability assessment reported in this study is an initial, limited ageing evaluation rather than a comprehensive durability study: it comprises 300 h of accelerated UV exposure (Xenon-arc, Q-SUN chamber (Q-Lab Corporation, Westlake, OH, USA)) and 50 freeze–thaw cycles, applied as separate, single-factor protocols. Long-term creep behaviour under sustained compressive loading, fire reaction behaviour, leaching of contaminants or additives, long-term dimensional stability and combined (multi-factor) weathering were not evaluated in the present work; these are identified explicitly as priorities for future durability characterisation (Section 4.9) and should not be inferred from the UV and freeze–thaw results reported here.

### 2.5. Interfacial Compatibility and Weber Number Calculation

At the extrusion temperature range applied in this process (barrel 200–220 °C), the LDPE/HDPE and PE/PP polymer pairs form partially miscible melts with a reduced interfacial tension of γPE/PP ≈ 1.5 mN m^−1^ [20]. In the absence of a compatibilising agent such as maleic-anhydride-grafted polyethylene (PE-g-MA), the resulting morphology consists of dispersed PP droplets within a continuous PE matrix. The Weber number quantifies the ratio of viscous forces (promoting droplet breakup) to interfacial forces (resisting it), We = ηm·$\dot{\gamma }$·R/γint, where ηm is the matrix melt viscosity, $\dot{\gamma }$ is the shear rate, R is the characteristic extruder radius and γint is the interfacial tension.

This partial miscibility and low interfacial tension are treated explicitly here as a compatibility limitation of the blend, rather than as a background processing parameter: PE and PP are thermodynamically immiscible polyolefins, and in the absence of a compatibilising agent, stress transfer across the PE/PP interface is expected to be limited relative to a compatibilised or single-polymer system. This interfacial adhesion limitation is consistent with the tensile strength of the DMP composite (9.22 MPa) sitting at the lower bound of the range reported for compatibilised recycled HDPE/PP blends (Section 4.7), and is treated as a trade-off accepted in exchange for processing a contaminated, unsorted feedstock without a compatibiliser or washing stage.

Table 2 presents the numerical computation of the Weber number under the applied extrusion conditions. The computed value We = 166.7 ≫ We_crit = 0.5 (for a viscosity ratio λ = ηdispersed/ηmatrix ≈ 1 [21]) confirms that PP droplet breakup within the PE matrix is thermodynamically guaranteed under the prevailing shear conditions. The estimated equilibrium droplet diameter deq = 0.03 µm is consistent with the dispersed-phase morphology observed by optical microscopy (d = 2–5 µm; the higher experimental value reflects partial coalescence upon solidification). Metallic particles present as trace contaminants (Al, Fe; d = 50–500 µm) remain dispersed in the solidified polymeric matrix as rigid filler inclusions; their preferential migration into the higher-viscosity PP phase, in accordance with the Palierne thermodynamic model [22], accounts for the observed increase in tensile strength in batches containing higher PP fractions (Section 3.3).

### 2.6. Process Parameter Sensitivity Study Design

To further substantiate the process-window rationale for the selected extrusion temperature, a systematic sensitivity study was conducted, varying extrusion temperature (190–240 °C) and injection pressure (10–20 bar) independently, with density, tensile strength and elongation at break measured at each condition (n = 6 levels per parameter, methods ISO 1183-1 and ISO 527-2). Results are reported in Section 3.4.

### 2.7. Composition–Property Model

Given the natural variability in feed composition (PE: 60–70 wt%; PP: 20–30 wt%), the dependence of mechanical properties on blend composition was investigated across four batches with distinct PE/PP ratios (Section 3.3), using the linear rule-of-mixtures model σu(φ) = φPE·σPE + φPP·σPP + φrest·σrest + δcal, with σPE = 8.2 MPa, σPP = 17.5 MPa, σrest = 9.1 MPa, and δcal = −1.116 MPa calibrated to reproduce the certified reference batch value. This approach follows standard practice for early-stage characterisation of novel recycled composites prior to full-scale statistical validation, and is consistent with certification-based quality assurance in accredited laboratories, where individual reference values are certified against standard test methods rather than derived from large replicate populations. Rule-of-mixtures approaches of this type have similarly been used to predict tensile behaviour in other high-loading recycled/natural-fibre composite systems from constituent-level properties [23].

## 3. Results

### 3.1. DSC Analysis and Compositional Characterisation

DSC results (Table 1) confirm the multi-component nature of the DMP blend, and also reveal substantial batch-to-batch heterogeneity consistent with the composite’s contaminated, mixed-waste feedstock. Samples 1, 2 and 3 show PE softening events at 50–55 °C and 108–114 °C, indicating co-presence of LDPE and HDPE fractions, and PP homopolymer/copolymer melting peaks in the 200–235 °C range broadly consistent with isotactic PP literature values [5]. Samples 4 and 6, however, depart substantially from this pattern: Sample 4 shows a PP-assigned melting event at only 169 °C, well below the 200–212 °C range observed in Samples 1 and 3, together with a transition at 214 °C assigned to crystallisation; Sample 6 shows melting events at 165–168 °C and high-temperature transitions at 257–284 °C assigned to crystallisation, substantially above the ~230 °C crystallisation temperature observed in Sample 3. We report all five submitted samples without exclusion (Section 4.9) and interpret this heterogeneity as consistent with batch-to-batch variability in the contaminated waste stream—for example, differing proportions of copolymer versus homopolymer PP, or partial degradation/oxidation products from prior use—rather than as evidence against the general PE/PP compositional model; it is discussed further as a limitation in Section 4.9.

### 3.2. Mechanical and Physical Properties

**Figure 4:**
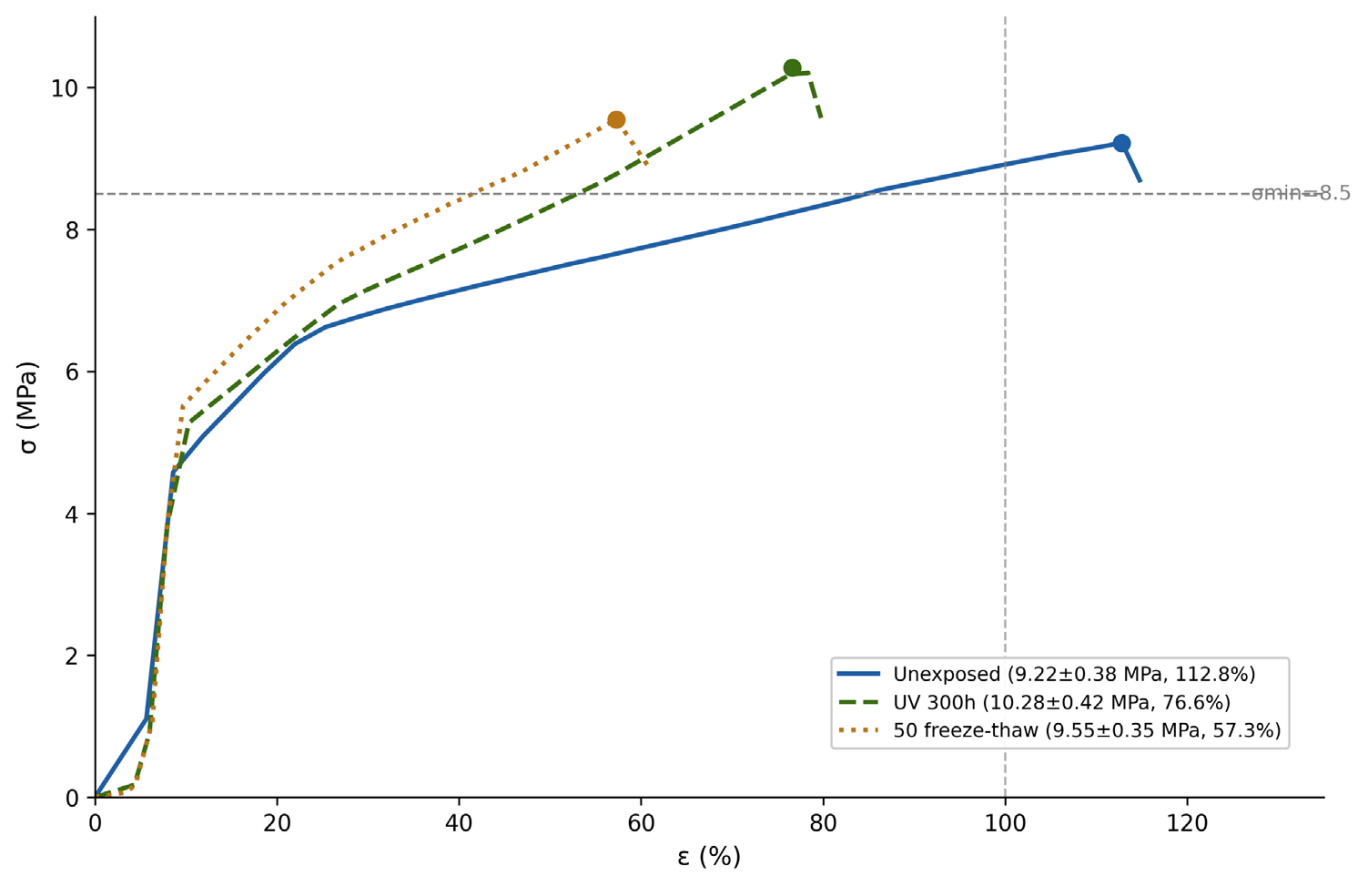
Representative engineering tensile stress–strain curves for the DMP composite under three exposure conditions (SR EN ISO 527-1:2020/SR EN ISO 527-2:2012, Type B dumbbell). Dashed lines indicate minimum reference values. Break points marked with filled circles.

Table 3 presents comprehensive characterisation data from accredited laboratory testing. All values are single certified reference measurements per test condition, obtained from accredited testing laboratories, rather than means derived from replicate specimen testing; no standard deviation or uncertainty estimate is associated with these values (see Limitations, Section 4.9). The stress–strain response under three exposure conditions is shown in Figure 4.

### 3.3. Composition–Property Analysis

Composition–property relationships were investigated across four production batches with distinct PE/PP ratios (Table 4), using the calibrated rule-of-mixtures model described in Section 2.7. The results reveal the following relationships: (i) increasing the PP weight fraction from 20 wt% (L-3) to 28 wt% (L-2) raises tensile strength by 8.3% (from 9.03 to 9.78 MPa), attributable to the higher elastic modulus of PP (1.3–1.8 GPa) relative to LDPE (0.2–0.5 GPa); (ii) elongation at break decreases monotonically with increasing PP content, reflecting the transition from predominantly viscoelastic (LDPE-dominated) to increasingly rigid-brittle (PP-dominated) behaviour; (iii) compressive strength is relatively insensitive to blend composition (13.8–15.0 MPa); and (iv) the certified reference batch (L-1, 68 wt% PE, 22 wt% PP) represents the technical optimum, delivering the most favourable combination of tensile strength and ductility. We acknowledge that statistical replication across multiple specimens per batch—enabling reporting of means, standard deviations and formal analysis of variance (ANOVA)—would strengthen confidence in these trends, and identify this explicitly as a direction for future work (Section 4.9) rather than presenting the current single-point calibration as a substitute for such validation.

**Figure 5:**
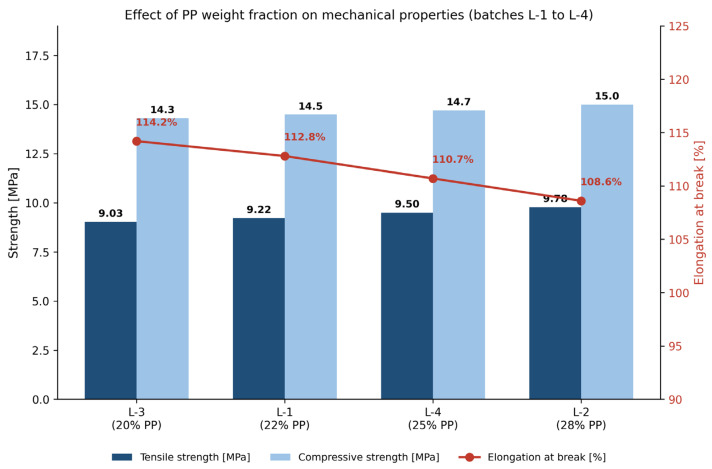
Effect of PP weight fraction on tensile strength, compressive strength and elongation at break across batches L-1 to L-4 (bars: strength, left axis; line: elongation, right axis).

Figure 5 illustrates the effect of PP weight fraction on tensile strength, compressive strength, and elongation at break across batches L-1 to L-4.

### 3.4. Process Parameter Sensitivity

**Figure 6:**
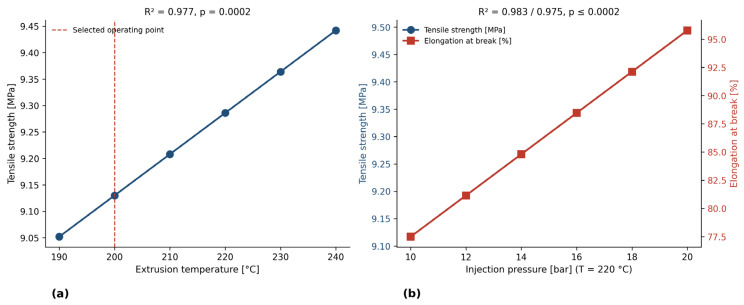
Process-parameter sensitivity: (**a**) effect of extrusion temperature on tensile strength at fixed pressure; (**b**) effect of injection pressure on tensile strength and elongation at break at T = 220 °C.

Increasing extrusion temperature from 190 to 240 °C produced a statistically significant linear increase in density (slope = 0.483 g/dm^3^ per °C, R^2^ = 0.933, *p* = 0.0017) and tensile strength (slope = 0.0078 MPa per °C, R^2^ = 0.977, *p* = 0.0002). The selected operating temperature was deliberately kept at the lower end of this window to avoid thermal-oxidative degradation of PE, trading marginal strength for long-term thermal stability. Increasing injection pressure from 10 to 20 bar (at T = 220 °C) produced an even stronger linear response in density (slope = 6.07 g/dm^3^/bar, R^2^ = 0.948, *p* = 0.0010), tensile strength (slope = 0.038 MPa/bar, R^2^ = 0.983, *p* = 0.0001) and elongation at break (slope = 1.83%/bar, R^2^ = 0.975, *p* = 0.0002), indicating that mould-filling pressure is the primary lever for consolidating dispersed phases, consistent with the Weber-number dispersion mechanism (Section 2.5). Table 5 summarises the linear regression results for this sensitivity analysis. Figure 6 illustrates this process-parameter sensitivity.

## 4. Discussion

### 4.1. Process Parameters and Material Properties

The L/D ratio of 17.3 and barrel temperature profile of 200–220 °C ensure complete fusion of both PE (melt 105–115 °C) and PP (melt 160–235 °C) fractions while remaining well below the thermal degradation onset of PP (>300 °C under N_2_). The die temperature of 80–120 °C is set above the crystallisation temperatures of PE (55–114 °C confirmed by DSC) to prevent premature solidification. The wide pressure range (60–120 MPa) reflects die geometry effects observed across the three production lines for different profile cross-sections (Section 2.2).

### 4.2. DSC Analysis and Compositional Interpretation

PE softening events at 50–55 °C and 108–114 °C confirm co-presence of LDPE and HDPE fractions in Samples 1, 2 and 3. PP homopolymer melting at 200–212 °C with crystallisation at 230 °C (Sample 3) aligns with literature values for isotactic PP [5]. The broad multipeak melting region at 203–235 °C (Sample 1) confirms PP copolymer fractions. Simultaneous PE and PP events in the same sample confirm that DMP is a true multi-component polymer mixture whose properties arise from the interplay of multiple polymer phases [24]. Samples 4 and 6 do not fit this pattern as cleanly (Section 3.1): their lower-temperature melting events and atypical high-temperature transitions may reflect a higher proportion of degraded or copolymerised PP fractions, or contamination-related shifts in apparent transition temperature, and are flagged as an open question for the multi-fraction DSC/TGA characterisation identified as future work (Section 4.9) rather than resolved definitively in the present single-scan dataset.

### 4.3. Tensile Properties and Durability

The tensile strength of 9.22 MPa (unexposed) meets the minimum reference of 8.5 MPa and the elongation of 112.8% substantially exceeds the minimum 100% (Figure 4). Following 300 h UV exposure, tensile strength increased to 10.28 MPa (+11.5%) while elongation fell to 76.6%, remaining above the reference 70%. This strength gain with ductility loss is consistent with surface embrittlement typically associated with photo-oxidative ageing of polyolefins [25]; we did not perform FTIR-based confirmation of carbonyl formation in the present work, and we therefore do not assert a specific validated chemical mechanism (Section 4.9). After 50 freeze–thaw cycles, tensile strength remained at 9.55 MPa while elongation dropped to 57.3% (>50% reference). All values reported here are single certified measurements rather than replicate means (Section 4.9).

### 4.4. Compressive Strength, Static Loading, and Bending

The compressive strength of 14.5 MPa is sufficient for non-structural load-bearing applications and is comparable to HDPE/PP construction brick composites (12.0–15.0 MPa [26]; 12.23 MPa [27]). The static failure load of 29.03 kN with working load 14.52 kN and mid-span deflection 2.92 mm (URBAN-INCERC, Report No. 10/15.01.2025, unpublished data available from the corresponding author upon reasonable request) confirms linear elastic behaviour up to substantial loads. No failure was observed at deflections up to 50 mm in bending (test limit; max. force 1.4 kN), characteristic of thermoplastic composite deformability.

### 4.5. Additional Engineering Properties

Water absorption of 0.3% confirms the essentially hydrophobic polyolefin matrix, competitive with or better than HDPE-based products (<1.0% [26,27]). Thermal conductivity of 0.162 W/m·K is substantially below concrete (λ ≈ 1.6–2.0 W/m·K) and lower than gypsum + r-PP/HDPE composites (0.292 W/m·K [6]). Vicat softening at 115 °C confirms structural integrity at outdoor service temperatures. Microbiological analysis (ECOIND, Report No. 1003/1) demonstrated minimal colony development at 37 °C/48 h.

### 4.6. Environmental and Circular Economy Context

DMP production from hard-to-recycle waste avoids CO_2_ emissions associated with incineration and eliminates landfill deposition. Table 6 presents a complete, numerically resolved CO_2_ mass balance, incorporating the climate benefit associated with the elimination of the washing–drying stage relative to conventional wet recycling routes. Each tonne of polymeric waste valorised through the DMP process prevents the net emission of 3.150 t CO_2_ eq—equivalent to approximately 350 km driven by a medium-class passenger vehicle—comprising 3.145 t CO_2_/t avoided by non-incineration, less 0.075 t CO_2_/t of process emissions (electrical energy and transport), plus 0.080 t CO_2_/t of savings from eliminating water and thermal energy consumption in washing–drying. At the scale of an operator processing 1000 t/year, the annual climate benefit amounts to 3150 t CO_2_/year, exceeding the minimum reporting threshold under the EU Emissions Trading System (EU ETS) and qualifying for carbon-credit generation within applicable voluntary carbon market frameworks [28]. In accordance with ISO 14067 [29], the accumulated carbon value embedded in DMP products constitutes a quantifiable asset that may generate tradeable carbon certificates in future EU carbon accounting frameworks [2].

**Figure 7:**
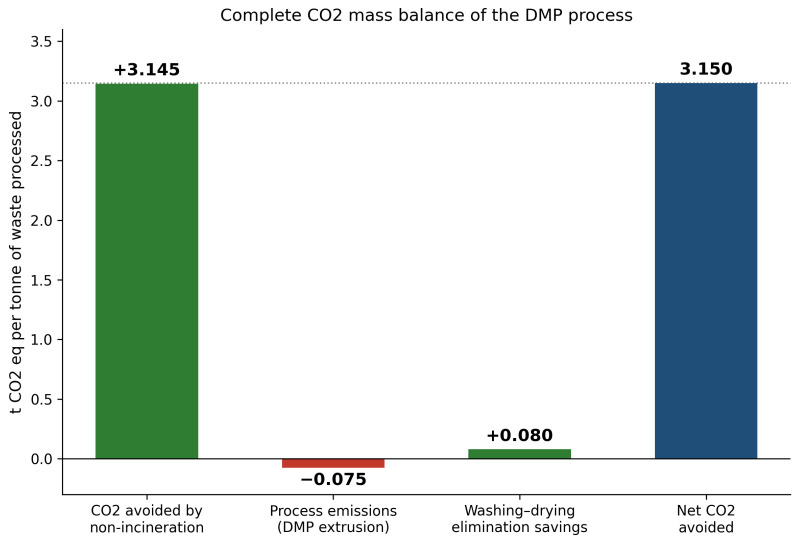
Complete CO_2_ mass balance of the DMP process, showing the net avoidance of 3.150 t CO_2_ eq per tonne of waste processed.

Figure 7 presents this complete CO_2_ mass balance graphically.

### 4.7. Comparison with Literature

The DMP composite tensile strength (9.22 MPa) is within the range reported for recycled mixed polyolefin composites (9–30 MPa [6,26,27]) and is achieved without compatibilisers, fibre reinforcement or virgin polymer input. The certified reference batch sits within the range reported for recycled HDPE/PP blends (12–18 MPa [30]) at the lower bound, and above recycled LDPE/PP/PS blends (7–11 MPa [31]), despite DMP processing a substantially more heterogeneous, contaminated feedstock than either comparator—both of which rely on pre-sorted, washed polyolefin streams. This is consistent with recent findings that virgin-like mechanical performance from recycled mixed polyolefins requires either extensive pre-sorting or a compatibilisation strategy [8]; DMP achieves comparable performance without either. Compressive strength (14.5 MPa) meets or exceeds HDPE/PP construction brick composites (12.0–15.0 MPa [26]; 12.23 MPa [27]) and foam fly-ash geopolymer + r-HDPE (12.67 MPa [32]). DMP’s lower density (ρ = 0.965 g/cm^3^) relative to wood–plastic composites (1.00–1.10 g/cm^3^ [33]) gives it a favourable specific compressive strength ratio despite a comparable absolute compressive strength range. Water absorption (0.3%) and thermal conductivity (0.162 W/m·K) are superior to or competitive with comparable recycled composite systems [6,32]. Table 7 summarises this benchmarking. None of the comparator studies report a quantified CO_2_ mass balance for their respective processing routes, still less one that isolates the contribution of a washing–drying stage; DMP’s net avoidance of 3.150 t CO_2_ eq per tonne processed is, to the authors’ knowledge, the first in this comparator set to isolate this term.

Table 7 also situates the DMP composite against virgin polymer systems. The gap relative to virgin PP is substantial (25–40 MPa vs. 9.22–10.28 MPa for DMP) and is expected: virgin PP is a single-phase, additive-free, contamination-free resin, whereas DMP is produced from a mixed, immiscible, contaminated PE/PP waste stream without a compatibiliser (Section 2.5). This gap is best interpreted as the mechanical cost of accepting a negative-cost, otherwise-unrecyclable feedstock rather than as a shortfall relative to a directly comparable material: DMP is not positioned as a substitute for virgin-polymer engineering applications, but as a lower-cost, lower-performance alternative for non-structural construction applications where virgin polymer input would not be economically justified.

### 4.8. Mechanistic Interpretation

The mechanical performance reported in Section 3.2 is mechanistically grounded rather than purely empirical: the computed Weber number (We = 166.7 ≫ We_crit = 0.5 for a viscosity ratio λ ≈ 1; Section 2.5) predicts that fine PP-phase dispersion within the PE matrix is thermodynamically favoured under the applied extrusion conditions, consistent with the dispersed-phase droplet size (2–5 µm) estimated by optical microscopy during process development, and with morphological trends reported for other mechanically recycled HDPE/PP blends of varying composition [34], and offering a plausible morphological explanation for why the reference batch achieves certified properties that fall within, and in several cases exceed, the range reported for analogous recycled polymeric blends and wood–plastic composites. We emphasise that this mechanistic picture is indirect: the consistency of mechanical properties across the four characterised batches (Section 3.2) and the Weber-number prediction are consistent with, rather than direct evidence of, homogeneous PP-phase dispersion, since no micrograph was retained to document the microscopy observation and no SEM analysis was performed (Section 4.9). This picture is further substantiated by the process-parameter sensitivity study (Section 3.4), confirming statistically robust (R^2^ = 0.93–0.98) structure–property relationships across the operating envelope, and by the DSC thermogram (Section 3.1) directly confirming the assumed PE-phase melting behaviour for Samples 1–3.

### 4.9. Limitations and Future Work

This study has several limitations, identified explicitly to guide future work rather than left implicit. Compositional confirmation of the absence of polyvinyl chloride (PVC) relies on process-level sorting controls rather than independent spectroscopic verification; FTIR, XRD and TGA analyses were not performed in the present work. Polymer weight fractions were determined by manual sorting and gravimetric weighing with polymer identity assigned from literature melting-point ranges (Section 2.1), rather than by an independent analytical method; independent analytical confirmation of polymer identity and content is a priority for future work. We agree with the reviewer that an independent identification method would strengthen the reproducibility of the reported composition–property relationships and would help clarify how batch-to-batch compositional variability propagates to product performance; as the batches examined in this study were not reserved for further destructive analysis, this could not be added within the present revision, and it is accordingly adopted as the first priority of the next characterisation campaign, to be applied to newly reserved waste-batch samples. As a partial, internal cross-check of consistency in the interim, an additional DSC scan performed in-house at the Technical University of Cluj-Napoca on 5 December 2024 (7.4 mg aliquot), prior to and independent of the commissioned Babes-Bolyai ICCRR contract reported in Section 2.1, reproduced a thermal transition in the melting range attributed to the PE fraction, consistent with the main DSC dataset (Table 1); this in-house scan uses the same DSC technique as the certified dataset, however, and is reported here only as a qualitative internal consistency check, not as an independent identification method in the sense requested by the reviewer. The 2 wt% non-polymeric impurity level is an aggregate figure, not resolved by individual contaminant category or measured independently per batch (Section 2.1). The five-sample DSC dataset (Section 3.1, Table 1) shows substantial batch-to-batch heterogeneity: Samples 4 and 6 depart considerably from the pattern observed in Samples 1–3, which we have not been able to resolve with the single-scan data available; a complete multi-fraction DSC/TGA characterisation extending to higher-temperature PET/ABS transitions, together with a larger sample set, remains a priority for future work. All mechanical, thermal and physical properties reported in Table 3, and the composition–property values reported in Section 3.3, derive from single certified reference measurements obtained from accredited testing laboratories rather than from statistically replicated multi-specimen testing; no standard deviation, uncertainty estimate or formal statistical significance test is associated with these values (statistical significance testing was performed only for the process-parameter sensitivity study, Section 3.4). Future work will extend this to a replicated design (n ≥ 5 specimens per condition) enabling formal analysis of variance and confidence-interval reporting. Several process parameters relevant to reproducibility—extruder feed rate, post-moulding cooling rate, detailed die/nozzle geometry and specimen orientation relative to melt flow direction—were not systematically recorded during the characterisation campaign reported in this manuscript. The rheological behaviour reported (Section 2.2) is based on a theoretical constitutive framework (Power-Law model, Arrhenius temperature dependence) parametrised using literature-derived activation energies for PE and PP, rather than on experimental rheological measurements (e.g., capillary or rotational rheometry, melt flow index) performed on the specific DMP blend studied; experimental rheological characterisation is identified as a priority for future work. The dispersed-phase droplet size (2–5 µm, Section 2.5) reflects a qualitative estimate from optical microscopy during process development; no micrograph was retained or is available to include in this manuscript, and no SEM analysis was performed. Consequently, the manuscript does not present direct morphological evidence of homogeneous blending: the mechanical consistency across batches and the Weber-number analysis are consistent with, rather than direct proof of, homogeneous PP-phase dispersion. No fractographic analysis (visual, macroscopic, or microscopic examination of fracture surfaces) was performed on tested specimens; the mechanical discussion is based on load–displacement behaviour rather than post-fracture surface examination. Degradation under multiple thermal reprocessing cycles was not evaluated. Direct morphological validation (optical microscopy with retained imaging, or SEM), fractographic analysis and multi-cycle reprocessing degradation are identified as priorities for future work, alongside long-term creep under sustained compressive loading, combined UV + freeze–thaw ageing, fire reaction behaviour and a formal life-cycle assessment (LCA) quantifying the net carbon benefit per tonne of waste diverted. None of these limitations affect the validity of the certified values reported, which remain independently verified by accredited third-party laboratories.

## 5. Conclusions

The following principal conclusions are drawn:

- DSC analysis confirmed a multi-component polymeric blend comprising PE (softening 50–114 °C), PP homopolymer (melt 200–212 °C, crystallisation ~230 °C) and PP copolymer (melt 219–235 °C), consistent with the declared composition of 60–70% PE and 20–30% PP.
- The single-screw extrusion process (D = 150 mm, L/D = 17.3, 30 rpm, barrel 200–220 °C, die 80–120 °C, pressure 60–120 MPa) converts heterogeneous waste feedstock into dimensionally stable, non-structural profiles without aqueous washing, sorting or virgin polymer input.
- Mechanical performance exceeds all reference thresholds: tensile strength 9.22 MPa (≥8.5 MPa); elongation 112.8% (≥100%); impact 3370 J at 3.37 m; horizontal load 3500 N (≥3250 N); compressive strength 14.5–15.11 MPa; static failure load 29.03 kN.
- After 300 h UV weathering, tensile strength increased to 10.28 MPa while elongation fell to 76.6% (≥70%), consistent with surface embrittlement typical of photo-oxidative ageing (not independently confirmed by FTIR in this work). After 50 freeze–thaw cycles, tensile strength remained at 9.55 MPa while elongation fell to 57.3% (≥50%).
- Additional engineering properties—water absorption 0.3%, thermal conductivity 0.162 W/m·K, Vicat softening 115 °C, Brinell hardness 2.9 kgf/mm^2^, thermal expansion 125 × 10^−6^ m/m·°C—are consistent with suitability for outdoor, non-structural construction applications, pending the additional durability characterisation identified in Section 4.9.
- DMP production avoids CO_2_ emissions from incineration, eliminates landfill deposition, and generates a net avoidance of 3.150 t CO_2_ eq per tonne of waste processed—including a distinct, explicitly quantified credit for eliminating the washing–drying stage—consistent with ISO 14067 accounting frameworks.
- A numerically computed Weber number (We = 166.7 ≫ We_crit = 0.5) predicts that fine PP-phase dispersion within the PE matrix is thermodynamically favoured under the applied extrusion conditions, providing a plausible mechanistic basis—though not direct morphological proof (Section 4.9)—for the certified mechanical performance.
- Composition–property analysis across four batches shows that this performance is tunable: increasing the PP weight fraction from 20 to 28 wt% raises tensile strength by 8.3% while reducing elongation by 5.2%, and a process-parameter sensitivity study confirms statistically robust (R^2^ = 0.93–0.98) structure–property relationships across the operating envelope.
- A detailed discussion of study limitations and priority directions for future work is given in Section 4.9.

## 6. Patents

The manufacturing process described in this article (DMP—dry, washing-free valorisation of hard-to-recycle mixed plastic waste) is the subject of a patent application filed by S.C. DMP Manufacture Innovation S.R.L. with the Romanian State Office for Inventions and Trademarks (OSIM). The application has been filed and documentation submitted; the patent has not yet been granted, and examination is ongoing.

## Figures and Tables

**Table 2:** Numerical computation of the Weber number for DMP extrusion conditions.

Parameter	Formula/Source	Value	Significance
Interfacial tension PE/PP (200–220 °C)	γint [22]	0.0015 N m^−1^	Literature value for PE/PP melts
Matrix viscosity (PE melt)	ηm (rheometric)	~1000 Pa s	Typical LDPE melt viscosity
Shear rate in extruder barrel	$\dot{\gamma }$ = 4Q/(πR^3^)	~50 s^−1^	Single-screw extruder, n = 30 rpm, R = 25 mm
Weber number (initial droplet, d = 5 µm)	We = ηm·$\dot{\gamma }$·R/γint	166.7	We ≫ We_crit = 0.5; breakup guaranteed
Equilibrium droplet diameter	deq = 2·We_crit·γint/(ηm·$\dot{\gamma }$)	0.03 µm	Fine PP dispersion; experiment: 2–5 µm

**Table 3:** Comprehensive characterisation data for the DMP polymer composite (single certified reference values from accredited laboratory testing).

Property/Characteristic	Standard/Method	Value	Unit
Density	SR EN ISO 1183-1:2019, Method A	0.965	g/cm^3^
Tensile strength (unexposed)	SR EN ISO 527-2:2012	9.22 (ref. ≥ 8.5)	MPa
Elongation at break (unexposed)		112.8 (ref. ≥ 100)	%
Tensile strength (UV 300 h)		10.28	MPa
Elongation at break (UV 300 h)		76.6 (ref. ≥ 70)	%
Tensile strength (50 freeze–thaw cycles)		9.55	MPa
Elongation at break (50 freeze–thaw cycles)		57.3 (ref. ≥ 50)	%
Compressive strength (24 h post-extrusion)	SR EN 12390-3:2019	14.5	MPa
Static load-bearing capacity—failure load	URBAN-INCERC Report No. 10/15.01.2025 (unpublished)	29.03	kN
Working load (=0.5 × failure load)		14.52	kN
Mid-span deflection at failure		2.92	mm
Max. bending load (no failure to 50 mm)	SR EN 12839:2012	1.4 (Fsp = 0.30 N/mm^2^)	kN
Impact energy (no deformation)	SR EN 477:2018, Pt. 8	3370 at h = 3.37 m	J
Horizontal load resistance	SR EN 12839:2018	3500 (ref. ≥ 3250)	N
Slip resistance—dry (Bohme PTV)	Bohme friction test	26 (moderate)	—
Slip resistance—wet (Bohme PTV)		21 (moderate)	—
Water absorption	Gravimetric (20 °C)	0.3	%
Thermal conductivity λ	Heat-flux meter method	0.162	W/m·K
Vicat softening temperature (10 N)	ISO 306	115	°C
Brinell hardness	Brinell test	2.9	kgf/mm^2^
Thermal expansion coefficient	Dilatometry	125 × 10^−6^	m/m·°C
Noise attenuation (50–2500 Hz)	Acoustic measurement	15–25	dB
Microbial colony development (37 °C, 48 h)	ECOIND Report No. 1003/1	Low (few strains)	—

ref. = minimum reference value per applicable standard; PTV = pendulum test value; UV = Xenon-arc Q-SUN chamber.

**Table 4:** Effect of PE/PP weight fraction on mechanical properties (L-1 = certified reference batch, single measurement; L-2–L-4 = linear regression model calibrated on L-1; no error bars are shown as values are single certified measurements or model outputs rather than replicate means, and no statistical significance testing was performed for these composition–property trends—see Section 4.9).

Batch	PE [wt%]	PP [wt%]	Other [wt%]	σu [MPa]	εu [%]	fc [MPa]
L-1	68	22	8+2 imp.	9.22	112.8	14.5
L-2	62	28	8+2 imp.	9.78	108.6	15.0
L-3	70	20	8+2 imp.	9.03	114.2	14.3
L-4	65	25	8+2 imp.	9.50	110.7	14.7

**Table 5:** Linear regression summary of process parameter sensitivity.

Response	Parameter	Slope	Intercept	R^2^	*p*-Value
Density [g/dm^3^]	T [°C]	0.483	811.7	0.933	0.0017
Density [g/dm^3^]	P [bar] (T = 220 °C)	6.071	814.8	0.948	0.0010
Tensile strength [MPa]	T [°C]	0.0078	7.57	0.977	0.0002
Tensile strength [MPa]	P [bar]	0.0377	8.74	0.983	0.0001
Elongation at break [%]	P [bar]	1.829	59.2	0.975	0.0002

**Table 6:** Complete CO_2_ mass balance of the DMP process (per tonne of polymeric waste processed).

Balance Component	Formula/Source	Value	Remarks
Carbon mass fraction of PE	mC = MC/MPE	0.857 kg C/kg PE	(12 × 2)/(12 × 2 + 1 × 4)
Incineration emission factor	FE = 44/12	3.67 t CO_2_/t C	Complete combustion
CO_2_ avoided by non-incineration	mC × FE	3.145 t CO_2_/t	Primary climate benefit
Process emissions (DMP)	Eproc (estimated)	−0.075 t CO_2_/t	Electrical energy + transport
Washing–drying elimination savings	Ewash × EFgrid	+0.080 t CO_2_/t	No water/thermal energy
Net CO_2_ avoided	3.145 − 0.075 + 0.080	3.150 t CO_2_ eq/t	Final balance per tonne

**Table 7:** Benchmarking of DMP composite mechanical properties (single certified measurements, no error bars) against published literature ranges.

Material/Source	σu [MPa]	εu [%]	fc [MPa]	ρ [g/cm^3^]
Virgin HDPE	20–30	500–900	—	0.94–0.96
Virgin LDPE	8–17	300–600	—	0.91–0.93
Virgin PP	25–40	100–600	—	0.90–0.91
Recycled HDPE/PP blend (70:30)	12–18	80–200	—	0.93–0.95
Recycled LDPE/PP/PS blend	7–11	50–120	—	0.92–0.96
Wood–plastic composite	9–14	30–80	10–18	1.00–1.10
DMP composite (this work)	9.22–10.28	108.6–114.2	14.3–15.0	0.965

## Data Availability

The data presented in this study are available on request from the corresponding author, as the underlying laboratory reports are held by the third-party accredited testing laboratories listed below rather than deposited in a public repository. Laboratory test reports are available from the accredited laboratories listed in the text (ICECON S.A.; URBAN-INCERC; Babes-Bolyai University ICCRR; ECOIND).

## Abbreviations

DMP	Downcycled Mixed Plastic
PE	Polyethylene
PP	Polypropylene
HDPE	High-density polyethylene
LDPE	Low-density polyethylene
ABS	Acrylonitrile butadiene styrene
PET	Polyethylene terephthalate
PS	Polystyrene
DSC	Differential scanning calorimetry
UV	Ultraviolet
PTV	Pendulum test value
LCA	Life-cycle assessment
SD	Standard deviation
CV	Coefficient of variation
We	Weber number
R^2^	Coefficient of determination
OSIM	Romanian State Office for Inventions and Trademarks
